# What Is Consciousness? Integrated Information vs. Inference

**DOI:** 10.3390/e23081032

**Published:** 2021-08-11

**Authors:** James E. Cooke

**Affiliations:** 1 Institute of Behavioural Neuroscience (IBN), University College London (UCL), London WC1H 0AP, UK; james.cooke@ucl.ac.uk; 2 Institute of Neuroscience, Trinity College Dublin, Dublin 2, Ireland

**Keywords:** consciousness, integrated information, inference, free energy principle, living mirror theory, explanatory gap, Markovian Monism

## Abstract

Any successful naturalistic account of consciousness must state what consciousness is, in terms that are compatible with the rest of our naturalistic descriptions of the world. Integrated Information Theory represents a pioneering attempt to do just this. This theory accounts for the core features of consciousness by holding that there is an equivalence between the phenomenal experience associated with a system and its intrinsic causal power. The proposal, however, fails to provide insight into the qualitative character of consciousness and, as a result of its proposed equivalence between consciousness and purely internal dynamics, into the intentional character of conscious perception. In recent years, an alternate group of theories has been proposed that claims consciousness to be equivalent to certain forms of inference. One such theory is the Living Mirror theory, which holds consciousness to be a form of inference performed by all living systems. The proposal of consciousness as inference overcomes the shortcomings of Integrated Information Theory, particularly in the case of conscious perception. A synthesis of these two perspectives can be reached by appreciating that conscious living systems are self-organising in nature. This mode of organization requires them to have a high level of integration. From this perspective, we can understand consciousness as being dependent on a system possessing non-trivial amounts of integrated information while holding that the process of inference performed by the system is the fact of consciousness itself.

## 1. Introduction

An explanatory gap currently exists in our scientific understanding of the world [1]. On one side of this gap are our third-person objective descriptions of physical phenomena. On the other side are our first-person subjective descriptions of our conscious experience. In order to close this gap, we need a theory of what consciousness is that can be stated in objective physical terms. At present, no such theory has been accepted by the philosophical and scientific mainstream.

What aspects of the physical world might correspond to consciousness? Due to the subjective character of consciousness, each of us can only know for certain that we ourselves are conscious. While this may appear to impose dramatic limits on our ability to make progress in this area, it also provides us with some evidence as to what kinds of systems can be conscious. It might be that the most fundamental units of what we are physically, the sub-atomic particles of the Standard Model of particle physics, are associated with consciousness [2,3,4,5]. As conscious living systems, we also know that a far-from-equilibrium open system can be conscious [6]. Finally, given the correlation between consciousness and brain activity, we can say that multicellular living systems with a centralised structure that performs complex computations (i.e., a brain) can be associated with consciousness [7]. It is outside the scope of this paper to consider this full range. Panpsychic and neuroscientific approaches will not be considered in favour of a focus on systems-level theories of consciousness that can be understood in terms of complexity theory [8]. Rather than offering exhaustive critiques of each of these theories, this paper will focus on a few key areas where these theories make different claims regarding certain important aspects of consciousness.

As lifeforms, we are characterised by a very particular systemic organisation. Arguably, our defining feature is that we behave in such a way as to perpetuate our form over time, resisting the tendency that we see in non-living systems to reach a state of equilibrium with the local environment. We are open systems that take in energy and dissipate it in such a way as to maintain the order of the system [6]. This feat is achieved through a process of self-creation or autopoiesis [9]. Successfully responding to the surrounding environment requires living systems to infer the distal causes of locally sensed signals. It has been proposed that this process may lay the foundations for consciousness in complex creatures like ourselves [10,11] or that it may be the basis of consciousness in all living systems [12]. This family of theories will be described here as inference-based theories. This inference rests on living systems being autopoietic systems that create themselves. As such, living systems are highly integrated. Integration here refers to the internal causal interactions of a system. For example, the feedforward circuitry of a digital camera where the activity of each photodiode is fully independent of the others shows no integration, while a recurrently connected neural network shows a higher level of integration. Integrated Information Theory (IIT) holds that this form of integration is what consciousness is, in physical terms [13,14,15,16,17].

How are we to assess whether these approaches can function as a successful account of the place of consciousness in the natural world? One approach is to provide empirical proof of the predictive power of the theory. These theories are indeed precise enough to generate predictions that can be tested experimentally [18]. Until we accrue enough evidence to assess the predictive power of these theories, we can assess them by considering their explanatory power. We can ask whether they account for features of consciousness in a way that is intuitively satisfying. While this approach is complicated by the potential fallibility of our intuitions and the fact that the natural world is under no obligation to be intuitively intelligible to us, the intuitive explanatory power of scientific theories inevitably plays a key role in how theories of emergent phenomena reach widespread acceptance.

## 2. Integrated Information Theory

IIT is a pioneering attempt to propose what consciousness could be at the level of the physical system [13,14,15,16,17]. The theory also attempts to account for why consciousness possesses certain features in light of this proposal. The description of consciousness that is offered in IIT is that consciousness is integrated information. Information should not be understood here as relating to Shannon information [19] but is instead the intrinsic cause–effect power of a system, the “differences that make a difference” [16]. A system that consists of fully independent components has no intrinsic cause–effect power, no differences in the activity of any component will make a difference to the rest of the system. The information associated with this system is, therefore, 0. Any system that does not consist of fully independent components influences its own states, moment to moment, and is associated with a non-zero amount of information. The precise structure of this influence amongst the components of the system is its cause–effect repertoire. The cause–effect repertoire that is associated with the system as a whole is considered irreducible or integrated; it cannot be defined fully by its component systems. Integrated information is the intrinsic cause–effect power of a system as a whole, over and above the contributions of its parts [17].

In IIT, consciousness is held to be equivalent to a specific aspect of the integrated information associated with a system, the Maximally Irreducible Conceptual Structure (MICS) [16]. A physical system is said to have intrinsic existence in this framework if it has a cause–effect structure. Systems with no cause–effect structure cannot be said to exist as systems, as there is no sense in which we can begin to describe systems that do not have an impact on the world and are not impacted by the world. The aspect of this cause–effect structure that is relevant to consciousness is the extent to which the system has cause–effect power over itself. There will be some redundancy and overlap in the cause–effect repertoire of the system but the system will contain a core repertoire that cannot be reduced further, and is said to be “maximally irreducible”. This maximally irreducible repertoire of cause–effect power is called a core concept. The same move of considering the maximally irreducible cause–effect structure is then made again at the level of these core concepts. This gives us the MICS that is held to be equivalent to consciousness. We have here a description of what consciousness is in third-person terms, and the theory uses this equivalence to provide insight into why consciousness has certain phenomenal properties [14].

Any successful theory of consciousness must account for the features that consciousness possesses. IIT attempts to achieve this by beginning with a set of phenomenological observations that are described as axioms. These features of consciousness are held to be self-evident.

(1) The first is intrinsic existence, consciousness exists from the system’s own perspective in a way that does not depend on its observation by others. This is a particularly significant feature of consciousness that needs to be accounted for. The inherent privacy of consciousness, its inaccessibility through third person methods, is the very feature of consciousness that makes it so challenging to investigate using the scientific method.

(2) Secondly, we have composition. Each conscious experience is composed of a particular set of component experiences, composed in a particular way.

(3) Next, we have the claim that consciousness is informative. Every conscious state specifies a particular experience to the exclusion of all others.

(4) The penultimate axiom holds that consciousness is integrated. Each conscious experience hangs together as a coherent whole that is fundamentally undivided.

(5) Finally, we have the exclusion axiom. The claim here is that while you can experience the world in multiple ways, these different experiences cannot happen simultaneously. Time may feel as if it is flowing quickly or slowly but one cannot experience both at the same time. The experience of one excludes the experience of the other. Relatedly, the contents of consciousness can only be a certain way to the exclusion of all other possible contents.

How does IIT account for the phenomenal features of consciousness that it describes in its axioms? It does so by suggesting a postulate for each axiom—a claim as to what the corresponding physical substrate of each phenomenal feature could be. The intrinsic existence postulate holds that a conscious physical system must exert cause–effect power on itself. It is this property that allows us to say that it exists intrinsically from its own side, as does consciousness. The composition of a conscious experience is attributed to the composition of this cause–effect structure. In IIT, the informative nature of conscious experience corresponds to the fact that the system will have a specific cause–effect structure at any one time and not at any other time. The integrated or holistic nature of consciousness is attributed to the requirement that the cause–effect structure be maximally irreducible. That is to say, it cannot be completely described by its subsystems, it necessarily forms a whole. Finally, the exclusive nature of a conscious experience is attributed to a single, dominant cause–effect structure that is associated with a specific spatial and temporal scale.

Does IIT provide a satisfactory account of its own phenomenal axioms? The key claim of IIT is that consciousness is equivalent to a particular set of cause–effect relationships, in which the system has an impact on itself. This satisfies the intrinsic existence axiom, the intuition that consciousness exists privately, from our own side. Intuitive accounts of the composition, information, and integration axioms follow naturally from this. Having a specific composition is a characteristic of both conscious experience and the cause–effect structure of a system, they are one particular way at any time to the exclusion of all other possibilities, and, for the structure to exist, it must be an integrated holistic whole—a characteristic of conscious experience. The exclusion postulate, however, follows less naturally. Not only is it unclear how the exclusion postulate relates to the exclusion axiom, it also introduces the problem that individual experiences are underdetermined [20]. Attempts have been made, however, to modify the theory to address this issue [21]. For more problems with IIT, see Section 5.

## 3. Inference-Based Theories

Another class of theories holds that consciousness is a form of biologically instantiated inference [10,11,12,22,23,24,25,26,27,28,29,30,31]. These theories start with the insight that biological systems can be understood as inferential systems. Under the framework of the free energy principle, living systems can be seen as needing to engage in a process of inference in order to survive [29,32,33,34,35,36]. In order to exist over time, living systems must assert a boundary with the world beyond [37,38]. The behaviour required to physically maintain this boundary is equivalent to the system instantiating Bayesian beliefs regarding the causes of the sensory stimulation that arrives at the system’s boundary [35]. Karl Friston and collaborators have made multiple proposals for how the complex forms of this inference process executed by the human brain might give rise to consciousness [10,11,22,23,24].

One such proposal is that consciousness is temporally deep inference with a sufficient amount of counterfactual depth [39,40] to enable the self-modelling of the organism [10]. While inferring the causes of locally sensed information can occur in an immediate and reflexive manner (temporally shallow inference); however, throughout evolution, the depth with which an organism can infer the causes of sensory information in time appears to have increased. In this proposal, this is the key cognitive ability that underpins the emergence of consciousness. Once an organism can model the world in this way, it can model a range of possible ways that the world could be (counterfactual depth), and it can also model its own influence on the world. In this picture, self-awareness is a crucial ingredient for phenomenal consciousness to exist in a system. This claim appears to be at odds with the well-documented phenomenon of phenomenal experience continuing in the absence of any experience of self [41,42,43,44]. A second proposal [22,23,24] is that consciousness is generated by the brainstem when internal homeostatic processing fails to predict the relevant internal signals. This particular proposal represents a specific neurobiological theory rather than a systems theory of consciousness, making it difficult to compare directly with IIT and other inference-based theories of consciousness.

A broader proposal regarding the relationship between biologically instantiated inference and consciousness has been suggested under the name Markovian Monism [11]. This ontological theory holds that consciousness must be understood as being dependent on the boundary maintaining dynamics of far-from-equilibrium steady state systems, like ourselves. These boundaries can be described statistically as Markov blankets, hence “Markovian Monism”. In this framework, consciousness is held to emerge gradually throughout evolution. Consciousness in Markovian Monism is defined as a “vague” concept. “Vagueness”, here, means that, in some systems, we cannot say whether or not consciousness exists. This inability to determine the consciousness of a system is not held to be due to methodological limitations, such limitations exist for assessing whether any system other than ourselves is conscious [45], but in principle. Consciousness, here, is not something that either does or does not exist but is instead vague. The theory, therefore, does not account for the presence or absence of subjectivity in specific systems. While this approach suggests a reasonable necessary condition for consciousness to emerge (a Markov blanket), we cannot use this theory to predict which systems are conscious and why that is. A theory that does do this is the Living Mirror theory [12].

The Living Mirror theory holds that consciousness is equivalent to the form of inference performed by all living systems in order to survive [12]. As a result, all living systems are conscious, even those without nervous systems. The proposal here is that all Markov-blanketed systems that maintain their form far-from-equilibrium must instantiate an interdependent framework of beliefs in the qualitative character of the world beyond their boundary that accounts for the sensory information that arrives at their boundary. This interdependent framework of beliefs in qualities is held to be an equivalent description of consciousness. The use of the term “belief” here should not be construed in the colloquial sense, as a complex cognitive phenomenon that presupposes consciousness or as a propositional belief that can be articulated using language. Instead, it should be understood as a system holding that the world is a certain way. A core claim of this theory is that holding such attitudes towards the world is the same thing as being conscious. The theory attempts to provide an intuitive explanation as to why consciousness exists; in living systems, beliefs in the character of the world beyond the system’s boundary are necessary for survival.

In the consciousness-as-inference framework, are we to consider Bayesian beliefs as equivalent to qualia? In the Living Mirror theory, this is not the case. Consciousness is held to be a process that exists in the embodied interaction of the organism with its environment. The representations inside the organism that we might call Bayesian beliefs are an important aspect; however, they are only one part. In this view, consciousness is a process, not a substance. By way of an example, running is not located in the lower limbs although they are a crucial part of this activity. The phenomenon of running exists where a system locomotes in a particular way through its environment; it cannot exist without the surface that the running is performed on, without gravity and so on. In the Living Mirror framework, the necessity of inference for biological survival is understood as the explanation for why and where consciousness exists-all living systems hold the world to be a certain way in order to survive. Consciousness exists where this activity occurs but it is not located in any particular part of the system.

The Living Mirror theory most directly accounts for the conscious perception of external phenomena but can also account for interoception and processes related to perception, such as learning and memory. In the case of learning and memory, the survival behaviour of the organism requires its representations to constantly adapt to the ever-changing environment. These survival dynamics result in persistent physical changes that underpin learning and memory. Due to these dynamics, no aspect of the physical process underlying consciousness is static, as the organism’s models of the world are constantly updated. Interoception must also be accounted for by any theory of consciousness. In the initial exposition of the Living Mirror theory [12], an account for exteroceptive perception was favoured, as this is straightforward to conceptualize the relationship between externally oriented perception and the detection of events that may affect the organism’s chances of survival. Such events, however, may also be detected through the measurement of internal states. If an organism is in a dangerously hot environment, this can be sensed by measuring both the external temperature or the internal temperature. Both the external and internal sources of information can be used to inform the boundary-maintaining behaviour of the organism, and thus both fit within the same framework.

Theories that hold individual cells to be conscious are faced with the challenge of accounting for how these multiple cellular consciousnesses in a multicellular organism relate to the single consciousness that we typically identify with [46,47]. One proposal is that communication between cells results in their consciousness being “turned over” to a centralised nervous system [46,48]. Under the framework of the Living Mirror theory, no such combination of consciousness is required. Whole organisms show the same dynamics as individual cells and, as a result, the organism can be considered a locus of consciousness as much as the individual cells [38]. Both exist alongside each other with no need for the multiple consciousnesses to interact.

The position that consciousness is coextensive with life is known as biopsychism [46,49,50,51,52,53]. Biopsychic theories offer a solution to a major challenge faced by neurobiological theories of consciousness that hold consciousness to require a nervous system. This is the ‘emergentist’ position [54], and it divides the living world into the conscious and the nonconscious. While the biopsychist perspective also holds consciousness to have emerged in the history of our universe, it does not hold that it emerged through the process of evolution. According to the evolutionary emergentist position, some organisms are not conscious but still have sensory-motor behaviours that involve information processing and inferential beliefs as defined here. Indeed, the purportedly nonconscious protozoans and bacteria do all these things. For the emergentist, there appears to be no principled place to draw a dividing line between the conscious and nonconscious along the continuum of organismal evolution. This is the discontinuity problem or emergentist’s dilemma, which also challenges the emergentist to account for what possible evolutionary advantages a phenomenal experience could confer over the functionally equivalent processes performed by nonconscious organisms [46,55,56]. In response to this challenge, emergentists have proposed advantages for consciousness, mostly along the lines that consciousness processes many sensory inputs into detailed maps of the environment to allow highly skilled navigation through complex, three-dimensional space [57,58,59,60]. However, organisms, such as paramecia and worms, that are designated nonconscious in this perspective, successfully navigate in space without such sensory maps, making this functional difference appear too minimal to account for the emergence of a dramatically new phenomenon, such as consciousness.

Unlike the process of gradual modification that occurs through evolution, the emergence of living systems represents a radically different phenomenon compared to non-living systems. The emergence of living systems is synonymous with the emergence of phenomena that are not found in non-living systems, such as self-organisation and metabolism [54]. For biopsychists, the leap from non-life to life represents a sufficiently dramatic change in the operation of parts of the physical world that a phenomenon, such as consciousness, could be brought into existence with this transition. Holding that consciousness emerged through evolution, on the other hand, raises the potentially insoluble hard-problem of consciousness [61]. If there are some organisms that we deem to be unconscious yet they are still capable of performing cognitive functions that we otherwise associate with consciousness, the requirement for phenomenal experience in the natural world becomes a mystery. Conversely, if we consider all life to be conscious, the hard problem disappears.

## 4. Can Inference-Based Theories Account for the Axioms of IIT?

Can inference-based theories account for the phenomenal axioms of IIT?

(1) The intrinsic existence of consciousness, the fact that it exists from its own side, can readily be accounted for by the autopoietic nature of living systems [9]. Living systems maintain their structure through a process of self creation, they are not created by an outside intelligence but instead self-create. It is precisely in order to achieve this feat that they must infer the character of the world beyond their boundary, as this is necessary to counteract forces that would mortally increase the entropy of the living system. The consciousness of the living system exists in service of the organism’s own survival, it need not be accessible from any other perspective.

(2) The composition axiom is accounted for by the fact that the inference performed by living systems relates to the affordances of the world beyond. At any moment, the single world outside of the organism presents an interconnected pattern of statistical regularities that it may need to infer. These patterns have a particular composition, and a behaviourally relevant subsection will be reflected in the composition of the conscious experience.

(3) This also accounts for the information axiom, as the particular scene sets the informative character of consciousness at any time.

(4) The integrated nature of consciousness is accounted for by the proposal in the Living Mirror theory [12] that the qualitative beliefs that constitute consciousness can only exist as an interdependent framework. As such, each bounded living system is associated with a single holistic conscious experience.

(5) The interdependence of this framework also accounts for the exclusion axiom. Each experience is the way it is in relation to all other possible experiences and so, as a result, each experience can be understood as being synonymous with the exclusion of all other experiences.

Friston, Wiese, and Hobson [11] also explored whether the axioms of IIT can be accounted for from the position of Markovian Monism and the Free Energy Principle on which it is based. Their findings agree closely with those given here, but I present them for their slightly different perspective and wording. They argued that the Bayesian mechanics that underlie consciousness in this perspective are

(1) intrinsic in the sense that they pertain to internal states of the system.

(2) They have a composition that arises from the statistical structure that exists in their interactions with the environment.

(3) Each conscious state is informative because it corresponds to a particular point on a statistical manifold that maps to the full range of possible experiences for that system.

(4) The dynamics underlying consciousness are integrated as each part of the system must impact the other internal states of the system in the regime that they describe.

(5) These dynamics exclude other possible beliefs when they instantiate a particular belief. Markov blankets can coexist at multiple spatiotemporal scales, however, meaning that multiple consciousnesses can exist within a single system. This is in sharp contrast to IIT where the principle of exclusion requires that a single consciousness exists in any integrated system.

One criticism of the ability of the inference perspective to account for the axioms of IIT pertains to the intrinsic existence axiom. If Bayesian beliefs are understood as models that an observer uses to describe how a system relates to its environment, then they do not have the property of existing from the organisms own side and cannot underwrite subjectivity, as argued by Albantakis [62]. My response to this issue is that it is not the models used by the scientist, however, that must become conscious, it is real, embodied systems. Models, such as Bayesian beliefs capture an aspect of the operation of living systems. Such models are not meaningful in the same way when applied to non-living systems. These models map the ability of an organism to hold that the world around itself is a certain way. As a result, we can say that it is like something for that system to exist in the world; whereas, there is no reason to believe this is the case for non-living systems.

## 5. Beyond the Axioms; Perception and the Qualitative Character of Consciousness

There are important aspects of consciousness that are not captured by the axioms of IIT. One is the qualitative character of consciousness. While the privacy of consciousness makes it impossible to study directly via traditional, third-person scientific methods, it is this qualitative character that makes consciousness so difficult to make sense of in the quantitative language of science [63,64]. Any scientific theory of consciousness must also account for the qualitative character of consciousness.

How does IIT account for this qualitative character? In IIT, consciousness is held to be equivalent to a structure of cause–effect repertoires through which the system impacts itself [16]. The particular qualitative character of an experience is held to be the same thing as the shape of the cause–effect structure in qualia space (Q). This is a multidimensional space in which each possible state in a system has a dimension and each subsystem specifies a point in this space. Directional relationships between subsystems can be represented in this space and it is the shape of these relationships, the Q-shape, that fully determines the qualitative character of a conscious experience [65]. This is the claim that attempts to close the gap between our quantitative description of the natural world and the existence of felt qualities [1]. Typically, scientific and philosophical claims of this kind are accepted, in part because they provide intuitive insight into the nature of the emergent phenomenon. Here, this would mean that describing qualitative experience as the structure of the cause–effect space would provide such insight. Why cause–effect structure should feel a certain way is not readily apparent, although attempts have been made to account for the qualitative character of certain experiences, such as the extendedness of space, within the framework of IIT [66]. Such attempts take the form of linking the structure of a substrate, such as the brain, with aspects of a conscious experience. As a result, they represent more detailed cases of the same core claim—that internal structure is what qualitative experience is.

IIT offers plausible explanations for many features of consciousness, yet it fails to provide an intuitive account of conscious perception. The contents of consciousness are often intentional, they refer to a world beyond the boundary of the organism [67]. As a result, our experiences do not simply signify themselves but instead evoke a world around us and an experience of ourselves within it [41]. If consciousness is the maximum of the internal causal dynamics of a system, why should this give rise to a conscious perception of the external world? For IIT, conscious perception is largely an internal affair, and percepts are accounted for as gestalts [8,65,68]. Why, then, do we consciously perceive an external world? According to IIT, internal structure comes to mirror external structure through a process called “matching” [15,69]. Matching is defined as the difference between two other terms, capture and mismatch. Capture measures how well a given system samples the statistical structure of its environment. Mismatch measures how well the system models this statistical structure. Subtracting mismatch from capture gives the level of matching exhibited in the system. This process of matching is argued to unfold through the adaptive operation of organisms, resulting in the internal structure of the system matching the structure of the environment that it must represent in order to survive.

Although this process may result in internal patterns that gain their structure from patterns in the external world, it does not account for how these become representations of those patterns that actually refer to something in the external world. The process of matching is akin to a process of resonance where two systems exhibit similar patterns. It does not, however, account for the intentional nature of the contents of consciousness. Under IIT, a system in an environment full of apples might come to have a conscious experience of an apple but it would not have the experience of perceiving an apple as existing beyond its boundary in an outside world. Despite the addition of the process of matching, consciousness itself is still a completely internal phenomenon in this framework. By focusing on the aspects of the system that are impacted by the system itself rather than the world surrounding it, IIT faces a major challenge in providing an intuitive account of why we perceive a world around us, why we feel that we see through our percepts to a world beyond. The focus on internal dynamics in IIT does allow it to account for the phenomenon of dreaming, however. In both IIT and the consciousness-as-inference framework, dreams can be easily accommodated by taking the “controlled hallucination” perspective on perception [70]. In this perspective, all conscious experience is internally generated (a “hallucination”) but in the waking state, the contents are “controlled” by incoming sensory input. In dreaming, conscious experience is decoupled from this sensory input; however, the ability of the system to generate a conscious experience remains.

In the Living Mirror theory, a different explanation for the qualitative character of conscious experiences is offered, one that is based in inference [12]. Here, the statistical structure of the organism’s niche that is relevant for survival is understood as existing implicitly before an organism is present to relate to them. In the presence of an organism, these statistical regularities become instantiated explicitly in the physical operation of the organism as inferential beliefs. The organism holds that the world is a certain way, as opposed to the other ways the organism could hold it to be. Potential food, for example, can be inferred to be more or less conducive to the organism’s flourishing if consumed and, as a result, a belief in the qualitative character of the food is held by the system. For example, rotting food does not smell “bad” due to some objective quantitative unit of “badness”, but rather the qualitative nature of the “bad smell” of rotting food results from its relation to our continued survival. Each belief stands in relation to all the other possible beliefs that the system could hold.

It is through this interdependence that such a framework of qualitative beliefs that could constitute consciousness can emerge out of a quantitative physical world. This is possible in the same way that a network can arise through the interactions of nodes that are themselves not networks. Interdependent phenomena can arise from components that do not possess their characteristics. This explanation of the qualitative character of consciousness directly accounts for why percepts should consist of specific qualities. On the other hand, IIT’s account of quality as internal cause–effect structure arguably does not provide a similarly intuitive account of qualitative character of consciousness. Ultimately, the differences between the two claims can be boiled down to the following statement, where a “feeling” is a qualitative experience. Are feelings internal structure, as in IIT, or are feelings attitudes to the state of the world in relation to yourself, as in the Living Mirror theory?

The strength of these inference-based theories, when compared with IIT, is that they directly account for the features of consciousness that IIT struggles with. Inference is understood to be a crucial aspect of perception [71]. Living systems infer distant causes of the sensory stimulation that occurs at their boundary, and this process, if understood as underpinning consciousness, readily accounts for the intentional character of consciousness [72]. The inferential beliefs that the system instantiates must refer to the world beyond in order for the system to survive. The perplexing, seemingly immaterial nature of consciousness can be accounted for by understanding it as a process of inference. If we consider the act of believing in a world outside of one’s boundaries to be the fact of consciousness itself, we can understand how it can simultaneously exist yet not be made of anything material.

Both IIT and the inference-based biopsychist perspective hold consciousness to be far more widespread than neurobiological theories, as they both hold that consciousness exists outside of nervous systems. In IIT, all systems that have any intrinsic causal structure are conscious. In the Living Mirror theory, consciousness is coextensive with life but cannot exist in non-living systems. If one judges the reasonableness of these claims from a skeptical stance in which we only know human beings to be conscious, the biopsychist perspective represents the more reasonable claim, as it only extends consciousness to other living systems. If, instead of emphasizing the role of the brain in the consciousness of the human, we instead consider experience to be a property of being an embodied living organism, then conferring consciousness on other embodied living organisms appears perfectly reasonable.

A similar argument could be used to support IIT. It is by virtue of our being an integrated system that we are conscious, then why not consider all integrated systems to be conscious? One way to differentiate these claims is to consider the fact that our consciousness is tied up with our biological survival. Pain typically feels bad, eating typically feels good, and so on. This feature of consciousness makes sense if consciousness is a biological phenomenon but must be accounted for if consciousness is grounded in our being an integrated system. The biological relevance of the contents of consciousness can give us a way to ground our intuitions around which systems are conscious. In IIT, certain simple arrangements of logic gates are deemed highly conscious, which violates these intuitions [73]. Taken together, the less extravagant claims of the widespread nature of consciousness in the biopsychist perspective and the biologically related contents of consciousness support the biopsychist stance over IIT.

As scientific theories of consciousness, the ultimate test of which perspective best accounts for consciousness will come from experimental work. IIT has generated a range of testable predictions that have been and continue to be explored experimentally [18,66,74]. As with IIT, the proposal of consciousness-as-inference has precise mathematical foundations that can be used to generate testable predictions, such as expecting perception to function in an approximately Bayesian manner [11,75]. This framework has also already generated testable predictions that have been explored through human neuroscience [76,77,78,79,80]. As we cannot measure the conscious experience of non-human animals, such experimental work will inevitably be confined to testing predictions for how human consciousness is structured and how this structure relates to the substrate of consciousness in the human. It is here that the relative merits of each of these perspectives will ultimately be decided.

## 6. Combining Integrated Information and Inference

Living systems are vastly more complex than non-living systems. They can be understood as self-creating, autopoietic systems that must act on themselves in order to perpetuate their form [9]. The only certain data point each of us has when it comes to the relationship between consciousness and its physical substrate is ourselves [45]. An individual human being represents a particularly complex form of living system. We are presented with the problem of which aspects of the living system might account for the phenomenon of consciousness. Is it the ability of the system to act on itself, its integrated information, or does this property merely play a necessary but supporting role for another process, that of inference?

In Markovian Monism, the kinds of statistical boundaries associated with living systems are held to be a necessary feature of the physical basis of consciousness [11]. We might consider integrated information in the same way. While it is not itself equivalent to consciousness, a physical system that is capable of instantiating the kind of inference that we might hold to be equivalent to consciousness must have a high level of integrated information, otherwise it would not be able to function as an autopoietic system. This perspective, in which integrated information plays a supporting role to a process of inference, accounts for why conscious systems, such as humans, are characterised by high levels of integrated information. It also accounts for the observation that systems that we do not intuitively expect to be particularly conscious, such as certain arrangements of logic gates, can have high levels of integrated information [73,81]. As mentioned above, IIT says that all systems with interacting parts are conscious if they form a maximum of integrated information. Such systems include protons, simple logic gates, and thermostats. This proposal has been criticised for being counterintuitive [82]. Being highly integrated is a necessary feature of a complex conscious system but it does not by itself, from this perspective, relate to consciousness.

Inference, on the other hand, appears to be a foundational feature of what it is to be the only kind of system that we know is conscious—a living system. Consciousness-as-inference accounts for why we consciously perceive a world around us and why consciousness is seemingly immaterial. If a consensus emerges around what form of inference is equivalent to consciousness, the final agreement will most likely come down to which proposal avoids insoluble philosophical problems, such as the hard problem of consciousness [61]. By presupposing that some living systems are not conscious, we create the discontinuity problem or emergentist’s dilemma, the problem of accounting for the presence of consciousness in some living systems as well as its absence in functionally similar nonconscious living systems. This also has the effect of creating the hard problem of consciousness. When we insist that nonconscious life exists, we then face the problem of stating what differentiates us from these nonconscious lifeforms. By taking a biopsychist perspective in which we hold all living systems to be conscious [49,50,51,52,53], we can avoid these problems and see the connection between the physical and experiential in the necessity of inferring the nature of the world beyond our boundaries in service of our physical survival.

## 7. Conclusions

The gap in our consensus understanding around the relationship between our description of the physical world and of consciousness must be closed by a theory that states what subjective experience is equivalent to, in objective terms. Integrated Information theory attempts to do this, and, while it offers intuitive explanations for many aspects of phenomenal experience, it fails to do so on the issue of intentional conscious perception. Inference-based theories, however, offer powerful, intuitive explanations for this aspect of conscious experience. Any complex self organizing system, like a human being, must be high in integrated information but we need not consider this aspect of the physical system to be the equivalent description of consciousness. Rather, we can see that the integrated information is a necessary requirement for a system to instantiate the forms of inference as is the case with self-organising living systems that operate far-from-equilibrium. If we hold consciousness to be equivalent to inference, we can account for many of the previously perplexing aspects of consciousness.

## Data Availability

No data was collected for this work.
